# Biological soil crusts determine the germination and growth of two exotic plants

**DOI:** 10.1002/ece3.3477

**Published:** 2017-10-10

**Authors:** Guang Song, Xinrong Li, Rong Hui

**Affiliations:** ^1^ Shapotou Desert Research and Experimental Station Northwest Institute of Eco‐Environment and Resources Chinese Academy of Science Lanzhou China; ^2^ University of Chinese Academy of Sciences Beijing China

**Keywords:** biological invasion, biological soil crust, biotic resistance, disturbance, exotic plant, germination percent

## Abstract

In arid and semiarid ecosystems, the potential threats of exotic invasive species are enhanced due to increasing human activities. Biological soil crusts (BSCs), acting as arid ecosystem engineers, may play an important role in preventing the establishment of exotic invasive plants. Our goal was to examine whether BSCs could inhibit the establishment of probable exotic plant species originating from adjacent grasslands located along the southeast edge of the Tengger Desert. In our study, we investigated the effects of three BSC types (cyanobacteria, lichen, and moss crusts) under two disturbance conditions (intact and disturbed) on the establishment of two exotic plant species (*Ceratoides latens* and *Setaria viridis*) using indoor experiments. We found both negative and positive effects of BSCs on the establishment of the two exotic plant species. Compared with the disturbed BSCs, the germination percentages of *C. latens* and *S. viridis* were reduced by 54% to 87% and 89% to 93%, respectively, in intact BSCs. In contrast, BSCs significantly promoted the height growth and aboveground biomass of the two exotic plant species (*p *<* *.05) by enhancing the soil water and nutrient availability for the exotic plants. Our results confirm that BSCs strongly suppress the rapid expansion of exotic plant populations by inhibiting germination of seed with big size or appendages and have a weak inhibitory effect on exotic plant with small and smooth seeds. This may decrease the threat of propagation of exotic species. In the meantime, BSCs promote the growth of a few successful engraftment seedlings, which increased the beta diversity. Our work suggests that better understanding the two opposing effects of BSCs on the establishment of exotic plant species in different growth stages (germination and growth) is important for maintaining the health and stability of revegetated regions.

## 1 INTRODUCTION

Biological soil crusts (BSCs) are photosynthetic and diazotrophic organisms that include cyanobacteria, green algae, lichens, and mosses and that have adapted to the environmental stresses of high light, temperature, and desiccation (Belnap & Lange, 2003; Belnap, Phillips, & Troxler, 2006; Li, Jia, Long, & Zerbe, 2005). In arid and semiarid ecosystems, cryptogamic species of BSCs are considered ecosystem pioneers because they colonize surface soils by closely integrating with particles of the soil surface (Belnap & Lange, 2003; Bu, Zhang, Zhang, & Wu, 2015; Karsten, Herburger, & Holzinger, 2014). Thus, BSCs aggregate and stabilize the soil surface against wind and water erosion (Belnap & Lange, 2003; Lan, Wu, Zhang, & Hu, 2015; Mazor, Kidron, Vonshak, & Abeliovich, 1996). They also strongly influence the hydrological cycle by altering the balance between water runoff and infiltration (Castillo‐Monroy, Maestre, Delgado‐Baquerizo, & Gallardo, 2010; Li, Kong, Tan, & Wang, 2007) and affect soil fertility by fixing carbon (C) and nitrogen (N) (Belnap & Lange, 2003; Elbert et al., 2012; Li, Zhang, Su, & Jia, 2012; Schulz, Mikhailyuk, Dreßler, Leinweber, & Karsten, 2016). Studies have indicated that BSCs provide available nutrients and appropriate habitats for vascular plants by improving the microenvironment, including the soil fertility, moisture, and temperature (Belnap & Lange, 2003; Bowker, Soliveres, & Maestre, 2010; Funk, Loydi, & Peter, 2014; Gao et al., 2014; Li et al., 2012). However, several other studies have demonstrated negative effects of BSCs on vascular plants (Hernandez & Sandquist, 2011; Morgan, 2006; Zhang, Wu, Zhang, & Zhang, 2010). Briggs and Morgan (2011) found that intact BSCs reduced the germination and establishment of the large‐seeded woody plant *Maireana excavata* in southeastern Australia. Notably, the negative effects of BSCs on the seedling establishment of certain species may increase habitat resistance to exotic plant invasions. For arid ecosystems therefore, another important ecological function of BSCs may be the inhibition of germination and establishment of exotic invasive vascular plants (Belnap & Lange, 2003; Hernandez & Sandquist, 2011).

Currently, biological invasions by exotic species are widely recognized as one of the most serious environmental problems because they threaten global biodiversity, cause enormous economic losses, and affect ecological security (Kelemen et al., 2016; Packer et al., 2017). Global land use change and human activities may increase disturbances and facilitate invasive exotic species by altering resource availability within communities and providing opportunities for exotic species to invade infertile and undisturbed habitats (Funk & Vitousek, 2007; Seebens et al., 2015). Indigenous diversity is facing many negative effects caused by the interaction of global change and biological invasion (Bradley, Wilcove, & Oppenheimer, 2010; Didham, Tylianakis, Gemmell, Rand, & Ewers, 2007; Dukes & Mooney, 1999; Eskelinen & Harrison, 2014). In addition, BSCs are highly vulnerable to disturbance and changing climatic conditions, and their recovery is a slow process (Belnap & Lange, 2003; Weber, Büdel, & Belnap, 2016). Disturbances in arid lands may reduce the ability of BSCs to inhibit the emergence and establishment of exotic plant species (Belnap & Lange, 2003; Beyschlag, Wittland, Jentsch, & Steinlein, 2008).Therefore, infertile and undisturbed habitats, such as those in arid lands, can no longer serve as important refuges for plant species diversity under increased human interference.

Arid lands, which cover more than one‐third of Earth's land surface, are areas with a severe and fragile ecological environment (Belnap, 2006; Maier et al., 2014). Arid ecosystems are also characterized by low vascular plant coverage because of low resource availability, low rates of nutrient turnover, and/or a limited ability of plants to acquire resources (e.g., extreme temperatures) (Adeel, 2008; Peel, Finlayson, & McMahon, 2007). These characteristics make arid ecosystems vulnerable to any disturbance, and the invasion of exotic plants is no exception. However, anthropogenic disturbances, such as agriculture, transportation, and industry, have facilitated the spreading and establishment of exotic species in barren arid habitats (Essl, Winter, & Pyšek, 2012; Pyšek et al., 2010). Recovery is difficult once arid ecosystems have been damaged by the invasion of exotic plants. For example, on the Caribbean coast of Colombia, the natural xeric habitats of the Peri‐Caribbean Arid Belt are classified as an endangered area partly because of the establishment of alien species (Chacón, Herrera, Flores, González, & Nassar, 2009). Therefore, early prediction and timely control of potential hazards related to exotic plant species are necessary (Essl et al., 2012; Li, Tian, Jia, Zhang, & Liu, 2010).

To our knowledge, studies focused on the relationship between BSCs and invasive plants have primarily investigated the threat of exotic plants to the composition and cover of BSCs (Belnap et al., 2006; Dettweiler‐Robinson, Bakker, & Grace, 2013; Peterson, 2013; Serpe, Roberts, Eldridge, & Rosentreter, 2013). However, few such studies have been specifically devoted to testing the inhibition of exotic plant emergence and establishment by BSCs. Because the observed effects of BSCs on vascular plants have been variable, ranging from negative to positive, it is worth evaluating specific situations, particularly if the vascular plant under consideration is or can potentially become an invasive weed.

In this study, we evaluate and precisely describe the effects of BSCs on soil moisture and nutrients, as well as the germination and growth of vascular plant species in the Shapotou regions at the southeastern edge of the Tengger Desert, which is characterized by a revegetation system with immobilized sand dunes in which BSCs are largely understudied. The revegetation system significantly improves the habitat for vascular plant colonization and promotes well‐developed BSCs (Li, Chen, & Yang, 2004; Li, Ma, Xiao, Wang, & Kim, 2004; Li et al., 2010; Su, Li, Zheng, & Huang, 2009). However, the system is still very fragile and prone to disturbances such as biological invasion (Li, Zhang, Wang, Liu, & Xiao, 1999). We predicted exotic plants species based on Baker's “ideal weed” method which lists biological characteristics favoring invasiveness (Baker, 1974, 1991) and is widely used to predict exotic invasive plants (Angert et al., 2011; Küster, Kühn, Bruelheide, & Klotz, 2008; Pyšek et al., 2010). Accordingly, we selected *Setaria viridis* (L.) Beauv. and *Ceratoides latens* Reveal et Holmgren as exotic vascular plants because of their biological characteristics, such as highly abundant propagules, rapid spreading, and tolerance of high light levels (Chambers & Norton, 1993; Jiang et al., 2011; Yi, Wang, Wu, & Zhang, 2003), consistent with the characteristics of an “ideal weed.” Moreover, *S. viridis* has been found in the study region and *C. latens* dominant on the adjacent desert steppe and could enter our study area due to increasing human activities (Li et al., 2010).

Using glasshouse germination and growth experiments, we tested the hypotheses that whether (1) intact BSCs will reduce seed germination of exotic plant species, leading to inhibition of the establishment of exotic plant species; (2) the growth of exotic plant species is facilitated by BSC‐induced increases in shallow soil moisture and nutrient content; (3) disturbances (e.g., livestock or anthropogenic activities) of BSCs may accelerate the emergence and establishment of exotic vascular plants.

## 2 MATERIALS AND METHODS

### 2.1 Site description

The study was conducted at Shapotou, which is located at the southeast fringe of the Tengger Desert in the Ningxia Hui Autonomous Region(37°33′N, 105°02′E) of northwestern China. The region is an ecotone between steppified desert and desertified steppe. Its zonal vegetation (i.e., the climax‐stage vegetation of this revegetated ecosystem) consists of *Reaumuria songarica* (Pall.) Maxim and *Salsola passerina* Bunge communities or *Stipa breviflora* Griseb communities, or a combination of the two community types (Shapotou Desert Research and Experiment Station Chinese Academy of Sciences, 1991, CAS 1991). Meteorological data, which were obtained from the Shapotou Desert Experimental Research Station, showed that the mean annual air temperature is 10.0°C with a minimum of −25.1°C in January and a maximum of 38.1°C in July. The mean annual precipitation is approximately 186 mm (1956‐2008), with approximately 80% occurring as rainfall from May to September. The annual duration of sunshine is 3,264 hr; mean annual wind velocity is 2.9 m/s; relative humidity is 45%; and the annual number of dust storm days is 59 (Shapotou Desert Research and Experiment Station Chinese Academy of Sciences, 1991, CAS 1991; Li et al., 2010).

A protective vegetation system that immobilizes sand on the dunes and ensures normal operation of the Baotou–Lanzhou railway located at the southeastern edge of the Tengger Desert. The protective vegetation system was established in 1956 by setting up a sand barrier and erecting a 1 m × 1 m straw checkerboard of 16 km long by 0.2 km wide on the south side and 0.5 km wide on the north side of the railway. Once the sand dunes had stabilized, several xerophytic shrub species, including *Artemisia ordosica*,* Caragana korshinskii*, and *Hedysarum scoparium*, were planted perpendicular to the main wind direction (Li et al., 2005).

After more than 50 years, this protective vegetation system promoted an increase in the cover of BSCs to more than 90%, which significantly improved the soil environment. The study area has been dominated by three predominant biological soil crust types, which developed successively from cyanobacteria into lichens and then mosses (Hu, Liu, & Song, 1998; Su et al., 2009; Lan et al., 2015). Lan et al. (2015)found that the development and succession of BSCs were promoted by improvement of ambient environmental conduction such as soil water, but succession could be halted by limited resources at a certain stage. Accordingly, three main successional stages of BSCs (cyanobacteria, lichen, and moss) are distributed at different positions on dunes, which are characterized by different microenvironments. Cyanobacteria crusts are mainly composed of filamentous cyanobacteria, green algae, and diatoms (Hu et al., 1998). Lichen crusts are mainly composed of *Collema tenax* (Sw.) Ach. and *Psora decipiens* (Ehrh.) Ach. and contain a minor amounts of *Xanthoparmelia deserborum* Hale. and *Diploschistes muscorum* (Scop.) R. Sant. Moss crusts are mainly composed of *Bryum argenteum* Hedw. and *Barbula ditrichoides* Broth. (Hu et al., 1998; Li, Chen, et al., 2004).

### 2.2 Soil samples and seeds collection

We collected three dominant soil crust types (cyanobacteria, lichens, and mosses) that occur in the Shapotou region. The cyanobacteria crusts were collected from dune tops; the lichen crusts were collected from windward and leeward slopes, and the moss crusts were collected from interdune depressions. Before sampling, the soil was slightly moistened to ensure that the BSCs were intact and less vulnerable to breakup during collection. Then, we used a cylindrical soil sampler with a 20 cm diameter and 20 cm depth to collect soil samples with and without (control soil treatment) crust. The respective samples were then cultured in separate pots under glasshouse conditions, at a constant temperature of 25‐33°C, with relative humidity ranging from 40% to 60%.

Adjacent to each core, we also collected a substrate sample from the top 5 cm of the soil with the crust layer removed for nutrient analyses (soil organic matter content total nitrogen and phosphorus content, and available nitrogen and phosphorus contents). The organic matter content was measured via the dichromate oxidation method; total nitrogen was measured using a micro‐Kjeldahl's method; total phosphorus was measured via the alkali diffusion method; available nitrogen was determined via the alkali diffusion method; and available phosphorus was measured using the sodium bicarbonate (NaHCO_3_) digestion‐Mo‐Sb colorimetry method (Bao, 2005).

**Figure 1:**
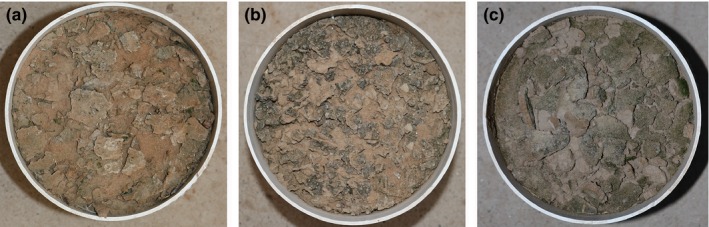
Photographs of disturbance of (a) cyanobacteria crust, (b) lichen crust and (c) moss crust

Additionally, to investigate the effects of BSC disturbance on the establishment of exotic plants, we devised two BSC treatments: intact and disturbed. In the intact treatment, the BSCs were left intact; in the disturbed treatment, the crusts were broken up using a stick (to simulate the effect of sheep hooves) so that the entire surface was evenly disturbed (Figure 1).

**Figure 2:**
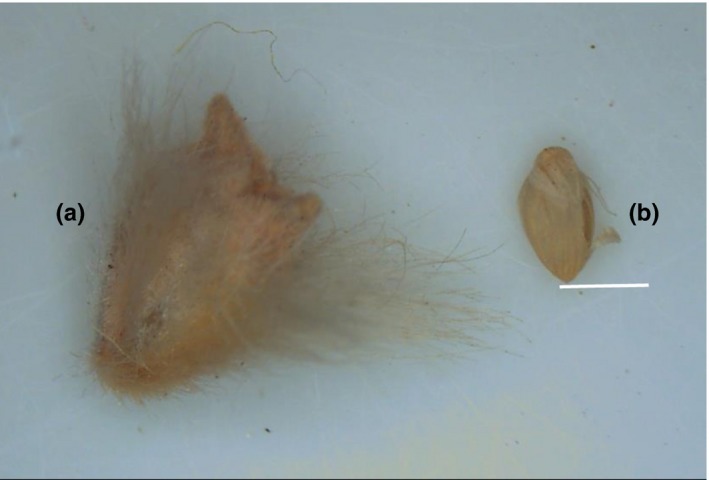
Morphology and size of C. lateens (a) and S. viridis (b) seeds. Scale bar is 1 mm. C. lateens seeds have appendages, and S. viridis seeds have a smooth surface

Seeds of the two exotic vascular plant species (Figure 2) were collected during the maturation stage (September to October and November to December in 2014, respectively) and kept at ambient temperature (average 5°C) in the laboratory until the experiments were conducted.

### 2.3 Experimental design

In 2015, we conducted a glasshouse experiment to estimate the effects of BSC types and disturbances on the seed germination, and growth of two exotic vascular plant species. The experimental design consisted of three types of BSC (cyanobacteria, lichens, and mosses), two levels of BSC disturbance (intact and disturbed), and a control treatment (bare soil). One hundred seeds of *C. latens* and *S. viridis* were placed on the surface of the intact and disturbed BSCs and bare soil. Five replicates were performed for each species and treatment, resulting in a total of 70 test pots. Prior to the glasshouse experiments, we tested the germination rates of seeds on moist filter paper, and the germination rates were 79% for *C. latens* and 86% for *S. viridis* (based on 100 seeds).

**Figure 3:**
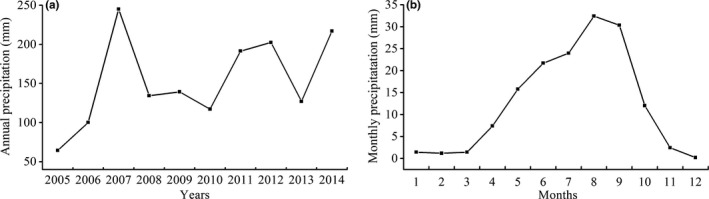
Annual precipitation (a) and monthly precipitation (b) of 2005‐2014 in the Shapotou region

We applied a moisture regime throughout the experiment: each pot received 9 mm per watering event every 10 days, consistent with the annual average precipitation (153 mm) and monthly precipitation from July to September (86 mm) of 2005‐2014 years in the Shapotou region (Figure 3). When the experiment began in early June 2015, we measured the soil moisture (m^3^·m^−3^) once a day in each pot using a time domain reflectometer (TDR, HH2‐Delta T Device Ltd., Burwell, UK). The TDR was calibrated before the experiment according to a new method for the measurement of sandy soil moisture (Chen, Chen, Xu, & Xu, 2010). The probe was inserted into the soil to a depth of 5–7 cm, and all readings were taken between 10:00 a.m., and 12:00 p.m. Every 2 days, the number of germinated seeds was counted, with a seed considered germinated when the radicle was visible. To minimize intraspecific competition, each species was randomly thinned to five individuals per plot after a month, and the seedling height of the five individual plants was measured (cm) from the soil surface every 5 days in each pot. In late September 2015, we harvested all surviving seedlings and measured the aboveground biomass.

### 2.4 Data analysis

The variance of each parameter (organic carbon, total phosphorus, total nitrogen, and available nitrogen and phosphorus contents) among the different experimental treatments was analyzed via a one‐way ANOVA. We used one‐way ANOVA to test the differences in soil moisture between intact and disturbed BSCs. The germination percentage, aboveground biomass, and plant height data were analyzed by mixed linear models, which are suitable for analyzing repeated‐measures data (Littell, Henry, & Ammerman, 1998). We treated the BSC types and disturbances as fixed factors. The significance of the differences between treatments was determined using an adjusted Tukey's test at *p *<* *.05. *C. latens* and *S. viridis* were analyzed in separate mixed linear models. Prior to these analyses, data were tested for assumptions of normality and homogeneity of variances and then sine‐ or log‐transformed when necessary. All the statistical analyses were performed using the software SPSS 20.0 for Windows (IBM SPSS Inc., Chicago, Illinois, USA).

## 3 RESULTS

### 3.1 Soil moisture and nutrients

Biological soil crusts were well colonized and developed during the successional process of sand‐binding vegetation in the Tengger Desert. Therefore, the soil organic matter content, total nitrogen content, total phosphorus content, and available nitrogen and phosphorus contents increased with succession from drift sand to cyanobacteria crust and then to lichen and moss crusts. Nevertheless, the disturbance of BSCs reduced the topsoil nutrient content. As shown in Table 1, the organic matter and soil nutrient contents were lower in the disturbed BSCs than in the intact BSCs, although these differences were not statistically significant.

**Table 1:** Comparison of soil physicochemical traits among the various Biological soil crusts (BSC) treatments

Soil treatments	Organic matter content (g/kg)	Total nitrogen (g/kg)	Total phosphorus (g/kg)	Available nitrogen (mg/kg)	Available phosphorus (mg/kg)
Bare soil	0.69 ± 0.06a	0.15 ± 0.002a	0.20 ± 0.007a	5.05 ± 0.012a	0.97 ± 0.014a
Disturbed cyanobacteria	6.50 ± 0.69b	0.13 ± 0.024a	0.27 ± 0.012b	8.57 ± 0.045b	2.36 ± 0.021b
Intact cyanobacteria	7.73 ± 0.66b	0.17 ± 0.030a	0.29 ± 0.015b	9.72 ± 0.024b	2.81 ± 0.043b
Disturbed lichen	6.97 ± 00.71b	0.31 ± 0.032b	0.30 ± 0.018c	15.26 ± 0.061c	3.23 ± 0.041b
Intact lichen	7.22 ± 0.38b	0.36 ± 0.026b	0.33 ± 0.014bc	16.35 ± 0.034c	4.12 ± 0.004c
Disturbed moss	12.50 ± 1.13c	0.48 ± 0.058c	0.36 ± 0.016c	17.1 ± 0.014d	5.27 ± 0.012d
Intact moss	13.49 ± 0.84c	0.51 ± 0.029c	0.37 ± 0.011c	18.02 ± 0.035d	5.85 ± 0.032d

For each measurement, four composite samples (each with triplicate subsamples from a single site) were evaluated (*n* = 4). The results are expressed as the mean±*SD*, and different lower case letters show significant differences in soil physicochemical traits among the BSC treatments.

**Figure 4:**
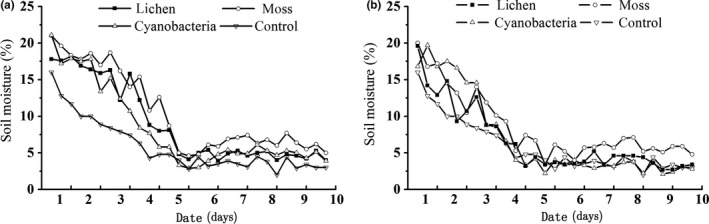
Changes in soil moisture after one water event under the intact (a) and disturbed (b) BSC

In general, soil moisture reached the lowest point at the sixth day after a water event and then remained relatively stable (Figure 4). However, the three BSC types showed different soil moisture retention capacities. Compared with the presence of sand, the presence of BSCs increased the topsoil water content, especially when the moss crust covered the topsoil. The moisture of soil covered by a moss crust was significantly higher than that of soil covered by a lichen crust, which in turn was significantly higher than that of soil covered by a cyanobacteria crust (*p *<* *.05).

In addition, disturbance of BSCs reduced the retention capacity for soil moisture. Analysis of variance showed that soil moisture was significantly different between intact and disturbed lichen crust (*p *<* *.05, *F *=* *4.804, *df *= 1) and moss crusts (*p *<* *.05, *F *=* *5.486, *df *= 1), whereas soil moisture showed no significant difference between intact and disturbed cyanobacteria crusts (*p *=* *.158, *F *=* *0.564, *df *= 1).

### 3.2 Germination

**Figure 5:**
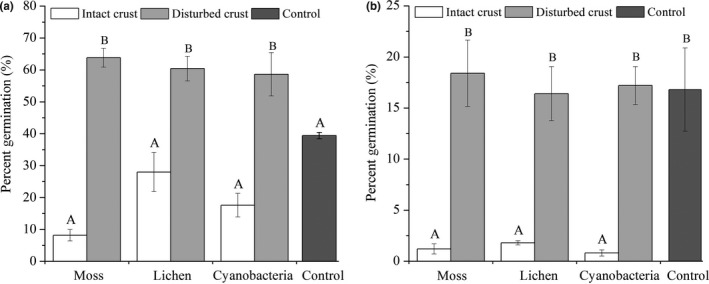
Germination percentages of S. viridis (a) and C. lateens (b) in the different experimental treatments. Values are means ± SE. Bars with different letters denote significant differences among different BSC types (ANOVA, P<0.05)

The seed morphology and main traits of *C. latens* and *S. viridis* are shown in Figure 2. Seeds of *C. latens* are wrapped in a utricle that is covered by hairs, whereas the seeds of *S. viridis* have a smooth surface and no appendages. Notably, the seed size of *C. latens* is three to four times larger than that of *S. viridis* (Figure 2), which significantly affected seed germination percentage in BSCs with the germination percentage of *S. viridis* seeds being significantly higher than that of *C. latens* (*p *<* *.05, *F *=* *20.015, *df *= 1). Compared with the disturbed BSCs, the intact BSCs significantly inhibited seed germination (*p *<* *.05, *F *=* *25.961, *df *= 1). As shown in Figure 5, the germination percentages of *C. latens* and *S. viridis*, respectively, were reduced by 87% and 93% with moss crusts, by 54% and 89% with lichen crusts, and by 70% and 95% with cyanobacteria crusts.

### 3.3 Plant height and biomass

**Figure 6:**
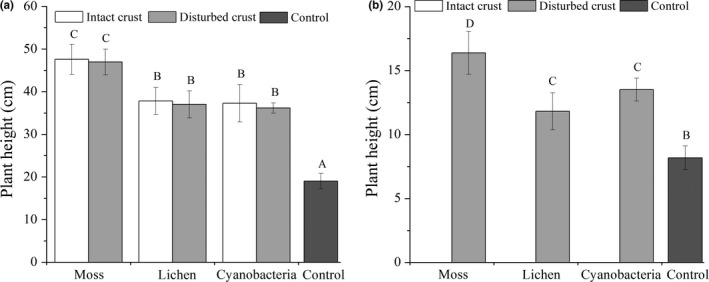
Plant heights of S. viridis (a) and C. lateens (b) in the different experimental treatments. Values are means ± SE. Bars with different letters denote significant differences among different BSCs treatments (ANOVA, P<0.05)

*S. viridis* showed no significant difference in plant height when comparing seeds planted in the intact BSCs with those in the disturbed BSCs (*p *>* *.05, *F *=* *1.359, *df *= 1, Figure 6a). However, heights of *S. viridis* planted in intact and disturbed BSCs were significantly greater than that in control soil treatments (*p *<* *.05, *F *=* *15.755, df = 1; *p *<* *.01, *F *=* *54.706, *df *= 1). Under disturbed conditions, *C. latens* plant height was greater in the moss crust soil than in the lichen and cyanobacteria crust soils (Figure 6b).

**Figure 7:**
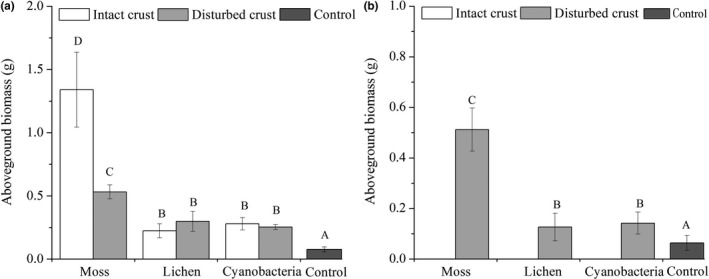
Biomass of S. viridis (a) and C. lateens (b) in the different experimental treatments. Values are means ± SE. Bars with different letters denote significant differences among different BSCs treatments (ANOVA, P<0.05)

On the disturbed crusts, the aboveground biomass of *S. viridis* and *C. latens* increased with the succession from drift sand to cyanobacteria and then to lichen and moss crust. Compared with that on drift sand, the aboveground biomass of *S. viridis* on moss, lichen, and cyanobacteria crusts increased by 600%, 300%, and 100%, respectively (Figure 7a), and the aboveground biomass of *C. latens* increased by 700%, 100%, and 100%, respectively (Figure 7b). On the intact crusts, the aboveground biomass of *S. viridis* showed a trend similar to that on disturbed BSCs. However, the larger size and hairy nature of *C. latens* seeds hindered their germination on intact BSCs and resulted in a lack of biomass data for the intact treatments (Figure 7b). However, the larger size and hairy nature of *C. latens* seeds hindered and delayed their germination on intact BSCs. Although several seeds germinated before the end of the experiment, their growth time was too short, and their height and biomass were therefore not compatible with the data from seedlings that had sufficient growth time. Therefore, in Figure 6b and Figure 7b, height and biomass data for the intact treatments are lacking.

## 4 DISCUSSION

Our study showed that disturbance and seed morphology had significantly different effects on seed germination in two plant species from adjacent grassland. The germination percentages of the seeds of *C. latens* and *S. viridis* did not differ significantly among the different types of BSCs. Nevertheless, compared with the disturbed BSCs, there were significant reductions in the germination percentages of both plant species under the intact BSC treatments. Analysis of the percentage of *S. viridis* germination also showed that there were no significant differences between intact crusts and bare soil (Figure 6a). However, this does not mean that sand can act as a natural barrier to exotic plants, as a large amount of sand indicates degeneration of the revegetation system (Li, Ma, et al., 2004, 2010). Therefore, a system characterized by sand and exotic plants is undesirable. These results demonstrate that intact BSCs can act as a natural biotic barrier to the establishment of dominant plant species from adjacent grassland, but disturbed BSCs would play an opposite role.

The germination percentages were significantly different between *C. latens* and *S. viridis*, perhaps related to differences in their seed morphology and size. Previous research has suggested that the morphology and size of plant seeds may affect germination on BSCs (Briggs & Morgan, 2011; Deines, Rosentreter, Eldridge, & Serpe, 2007; Li et al., 2005; Morgan, 2006). In this study, the larger size and hairy nature of *C. latens* seeds hindered the seeds from entering cracks or depressions in intact BSCs. Therefore, the seeds could not reach the relatively humid environment required for germination. In contrast, the smooth and smaller *S. viridis* seeds more easily reached appropriate germination environments.

The seeds of the two studied plant species do not have a self‐burial structure (e.g., hygroscopic awns) that facilitates the penetration of seeds into intact BSCs. Therefore, the seeds of *C. latens* and *S. viridis* can only enter the crustose soil via external disturbances, such as grazing, livestock, or anthropogenic activities. Increasing disturbances may assist the spread of plant species because such disturbances damage the biotic component (e.g., BSCs) and alleviate the “biotic resistance” function of the local ecosystem (Hernandez & Sandquist, 2011; Levine, Adler, & Yelenik, 2004; Shinneman, Baker, & Lyon, 2008). Previous studies have suggested that disturbance might enhance the germination of plant species (Belnap & Lange, 2003; Hernandez & Sandquist, 2011; Li et al., 2005). Our results also showed that the germination percentages of the two plant species on the disturbed BSCs were significantly higher than those on the intact BSCs (Figure 5). In a controlled environment, Serpe et al. (2013) observed that intact lichen crusts have negative effects on the establishment of *Bromus tectorum* and *Vulpia microstachys* despite the germination of seeds, and they attributed the lack of establishment to the fact that young roots were not able to penetrate the lichen thallus, causing the necrosis of root tips. The presence of intact BSCs limited the germination of seeds from adjacent grassland; this may inhibit the establishment and spread of these plant species in the study region.

Several reports have indicated that BSCs support vascular plants by altering the distribution of soil nutrient contents (e.g., carbon and phosphorous) and moisture (Castillo‐Monroy et al., 2010; Elbert et al., 2012; Li et al., 2007, 2012). The analysis of soil nutrients showed that the development of BSCs significantly increased the soil contents of organic matter, available nitrogen, and total phosphorus, and the levels of soil nutrients were significantly higher under the moss crusts than the other treatments (Table 1). We also found that exotic plant biomass significantly increased with the development of BSCs (Figure 7). It is evident from our results that soil nutrient availability is an important driver of plants biomass. This result is also supported by field evidence from hot and temperate deserts; Redfield, Barns, Belnap, Daane, and Kuske (2002) and Li et al. (2012) demonstrated that later‐successional lichen and moss crusts presented a higher protective ability and better topsoil nutrient and water retention capabilities than the early‐successional cyanobacteria crust. Accordingly, our tested species could obtain increasing amounts of available nutrients with the succession from bare sand to cyanobacteria crust and then to lichen and moss crusts. In addition, studies have reported that BSCs favor nutrient uptake by vascular plants and facilitate biomass accumulation in plant tissues (Harper & Belnap, 2001; Langhans, Storm, & Schwabe, 2009; Liu, Tao, Qiu, Zhang, & Zhang, 2013; Thiet, Doshas, & Smith, 2014). Our results also demonstrated that the plant height growth and aboveground biomass were much higher with the BSCs than with bare sand, especially the aboveground biomass, which was higher with the moss crusts than with the other substrates.

Another factor explaining the increased height growth and biomass of these exotic plant species in the presence of BSCs is the redistribution of water in the soil covered by BSCs. Numerous studies have shown that BSCs reduce infiltration because cryptogamic species of BSCs prolong water retention and duration in the topsoil (Belnap, Welter, Grimm, Barger, & Ludwig, 2005; Chamizo, Cantón, Domingo, & Belnap, 2013; Kidron, Yaalon, & Vonshak, 1999; Li, Ma, et al., 2004). In addition, previous studies in the Shapotou region have shown that the development of BSCs on the soil surface improves development of topsoil, including increases in fine soil particles, organic matter, and porosity (Li, Ma, et al., 2004; Su et al., 2009; Wang, Young, Yu, Li, & Zhang, 2007). The improvement of soil structure allows for higher water holding capacity, higher water availability, and higher porosity. As shown in Figure 4, in the early stages after water events, both intact and disturbed BSCs can maintain higher soil water contents than the control treatments. However, the soil water contents in the BSCs were lower than in the control at the later stages after water events. These findings can be explained by the physical and biological features of BSCs, which form a “protective coat” on the soil surface. The “protective coat” prolongs water retention in the topsoil by decreasing the porosity of the crust layer, and cryptogamic species, such as lichens and mosses, can absorb large amounts of water and limit the depth of water infiltration (Atwood & Krannitz, 1999; Li et al., 2010). Therefore, soil moisture is maintained in shallow layers by BSCs, improving the duration and availability of moisture for *S. viridis*, which has shallow roots, and the seedlings of *C. latens*. Soil water is the key abiotic limiting factor in desert areas, and increases in topsoil moisture determine increases biomass and height of exotic plants as BSCs develop (Figure 7). The results of this work showed that BSCs have certain promoting effects on the survival exotic plants.

## 5 CONCLUSIONS

Further economic development and increasing human activities in the arid regions at the edge of the Tengger Desert may lead to severe disturbances and increases in the exchange of plant species between adjacent ecosystems (Essl et al., 2012; Pyšek et al., 2010). These changes might increase the potential threat of biological invasion into the desert ecosystems. In glasshouse experiments, the presence of BSCs, which act as “ecosystem engineers” in arid ecosystems, was observed to significantly reduce the germination of seed with big size or appendages of exotic plant species, and they have a weak inhibitory effect on exotic plant with small and smooth seeds. On the other hand, we also found that BSCs could promote the growth of seedlings because they provide available nutrients and appropriate habitats for these exotic plants. In general, the dual characteristics of BSCs maintain exotic plant populations at low levels by inhibiting their rapid expansion, and these exotic plant species may be regarded as contributing to beta‐biodiversity. Therefore, the threat of exotic species may be decreased, and the beta diversity of plant species may increase, promoting the health and stability of revegetated regions.

## CONFLICT OF INTEREST

All of the authors declare they have no conflict of interest regarding this research.

## AUTHOR CONTRIBUTION

X.L., R.H., and G.S. designed research; G.S. and R.H. performed research; G.S. analyzed data; G.S., R.H., and X.L. wrote the manuscript.
